# Alterations in the Hypothalamic–Pituitary–Adrenal Axis as a Response to Experimental Autoimmune Encephalomyelitis in *Dark Agouti* Rats of Both Sexes

**DOI:** 10.3390/biom14081020

**Published:** 2024-08-17

**Authors:** Ana Milosevic, Katarina Milosevic, Anica Zivkovic, Irena Lavrnja, Danijela Savic, Ivana Bjelobaba, Marija M. Janjic

**Affiliations:** Department for Neurobiology, Institute for Biological Research “Sinisa Stankovic”, National Institute of Republic of Serbia, University of Belgrade, 11060 Belgrade, Serbia; ana.milosevic@ibiss.bg.ac.rs (A.M.); katarina.tesovic@ibiss.bg.ac.rs (K.M.); anica.zivkovic@ibiss.bg.ac.rs (A.Z.); irenam@ibiss.bg.ac.rs (I.L.); danisto@ibiss.bg.ac.rs (D.S.); ivana.bjelobaba@ibiss.bg.ac.rs (I.B.)

**Keywords:** EAE, multiple sclerosis, HPA axis, corticosterone, progesterone

## Abstract

Multiple sclerosis (MS) is a chronic inflammatory disease that affects the central nervous system, usually diagnosed during the reproductive period. Both MS and its commonly used animal model, experimental autoimmune encephalomyelitis (EAE), exhibit sex-specific features regarding disease progression and disturbances in the neuroendocrine and endocrine systems. This study investigates the hypothalamic–pituitary–adrenal (HPA) axis response of male and female *Dark Agouti* rats during EAE. At the onset of EAE, *Crh* expression in the hypothalamus of both sexes is decreased, while males show reduced plasma adrenocorticotropic hormone levels. Adrenal gland activity is increased during EAE in both males and females, as evidenced by enlarged adrenal glands and increased StAR gene and protein expression. However, only male rats show increased serum and adrenal corticosterone levels, and an increased volume of the adrenal cortex. Adrenal 3β-HSD protein and progesterone levels are elevated in males only. Serum progesterone levels of male rats are also increased, although testicular progesterone levels are decreased during the disease, implying that the adrenal gland is the source of elevated serum progesterone levels in males. Our results demonstrate a sex difference in the response of the HPA axis at the adrenal level, with male rats showing a more pronounced induction during EAE.

## 1. Introduction

Multiple sclerosis (MS) is a chronic inflammatory disease of the central nervous system (CNS) that affects over two million people worldwide. It is usually diagnosed in the reproductive period between the ages of 20 and 40 [1]. Multiple sclerosis is predominantly diagnosed in females, with only 25% of MS patients being men. Conversely, men have a poorer prognosis—they present with worse symptoms, advance faster to secondary progressive MS, and develop primary progressive MS more often than women [2]. Possible explanations for the different prevalence and clinical presentation of autoimmune diseases include differences in sex chromosomes and sex hormones [3] and sex-specific glucocorticoid actions [4]. Experimental autoimmune encephalomyelitis (EAE) is the best-characterized and most commonly used animal model to study MS. We have previously shown sex differences in the regulation of the hypothalamic–pituitary–gonadal (HPG) axis during EAE due to hypothalamic inflammation [5,6,7]. The interdependency between the hypothalamic–pituitary–adrenal (HPA) and HPG axes and their interference is well recognized. Glucocorticoids impact the HPG axis at multiple levels, and in the context of MS and EAE, disruption of the HPA axis has been reported [8,9,10]. In EAE, glucocorticoid administration improves clinical symptoms of neuroinflammation in a dose-dependent manner [11]. However, sex differences in HPA axis function are crucial as they influence the predisposition to therapeutic responses following glucocorticoid treatment [12]. This may be of particular relevance for MS because the standard treatment of acute relapses with high doses of corticosteroids is often associated with side effects [13]. Considering all these points, it is essential to understand the status of the HPA axis in MS and EAE in both sexes.

The regulation of the HPA axis is a tightly controlled process involving various neural and endocrine factors. Glucocorticoid biosynthesis is regulated primarily by adrenocorticotropic hormone (ACTH), which is secreted by the anterior pituitary in response to corticotropin-releasing hormone (CRH) and arginine-vasopressin (AVP), the neuropeptides secreted by the paraventricular nucleus of the hypothalamus in response to stress. Elevated levels of circulating glucocorticoids inhibit HPA activity at the level of the hypothalamus and pituitary, but the HPA axis is also subject to glucocorticoid-independent regulation [14].

The mammalian adrenal glands consist of two parts, the cortex and the medulla, which differ in origin, structure, and function. The adrenal medulla secretes catecholamines, epinephrine, and norepinephrine. The adrenal cortex is divided into three zones, the glomerulosa (zG), the fasciculata (zF), and the reticularis (zR), which secrete steroid hormones—mineralocorticoids, glucocorticoids, and androgens, respectively [15]. In contrast to human adrenal glands, rats exhibit low expression of 17α-hydroxylase/17–20 lyase [16], resulting in minimal synthesis of androgens in rat adrenal glands [17]. Consequently, in rats, corticosterone serves as the primary glucocorticoid, whereas humans predominantly produce cortisol.

The synthesis of mineralocorticoids and glucocorticoids occurs in several enzymatic steps from the precursor cholesterol. In the rodent adrenal gland, cholesterol esters, derived from high-density lipoproteins and released via the scavenger receptor class B type I (SR-BI), are the dominant cholesterol source for steroidogenesis [18]. The translocation of cholesterol from the outer to the inner mitochondrial membrane is a hormone-sensitive and rate-limiting step of steroidogenesis, mediated by steroidogenic acute regulatory protein (StAR) [19]. The first enzymatic step involves the conversion of cholesterol to pregnenolone by the cholesterol side-chain cleavage enzyme cytochrome P450 (P450scc). Pregnenolone is converted to progesterone by the enzyme 3β-hydroxysteroid dehydrogenase (3β-HSD). In rats, progesterone is converted to deoxycorticosterone (DOC) by the enzyme 21-hydroxylase cytochrome P450 (CYP21A1). Finally, in the zG, DOC is converted to aldosterone by the enzyme aldosterone synthase cytochrome P450 (CYP11B2), whereas in the zF/zR, it is converted to corticosterone by the enzyme 11β-hydroxylase cytochrome P450 (CYP11B1) [20]. The enzyme catalyzing the conversions between the active glucocorticoids, cortisol in humans and corticosterone in rats, and their inactive forms, cortisone and 11-dehydrocorticosterone, respectively, is 11β-hydroxysteroid dehydrogenase (11β-HSD) [21]. In rat adrenal glands, two isoforms of this enzyme were found: 11β-HSD1, which catalyzes the conversion from corticosterone to 11-dehydrocorticosterone, and 11β-HSD2, which catalyzes the reverse reaction [22].

To gain insight into sex differences in MS using an animal model, this study aimed to investigate possible sex-specific differences in the HPA axis response during EAE in rats. To achieve this, we performed EAE experiments in *Dark Agouti* (DA) rats of both sexes to evaluate the symptoms, hypothalamic mRNA levels of CRH and AVP, and plasma ACTH levels during the course of the disease. In addition, we evaluated adrenal gland morphology and examined gene and protein expression of adrenal enzymes and proteins involved in corticosterone production and measured serum, adrenal, and testicular levels of corticosterone and/or its precursor progesterone. We demonstrated that male rats develop a more severe acute monophasic EAE than female rats. There was a paradoxical decrease in serum ACTH levels and CRH mRNA levels in the hypothalamus of males at the onset of EAE. At the same time, changes in adrenal morphology were observed in males only during the disease. The results also showed an induction of StAR in both sexes and an upregulation of 3β-HSD in male adrenal glands, in line with a pronounced increase in male serum corticosterone levels. Depending on sex, the rise in serum progesterone had a different origin. Overall, the results presented here show that male rats exhibit a more robust response of the adrenal gland during EAE.

## 2. Materials and Methods

### 2.1. Animals

The experiments were performed using male and female DA strain rats, 9–12 weeks old, bred at the experimental animals breeding facility of the Institute for Biological Research “Sinisa Stankovic”. The rats were randomly distributed to cages and housed 4 per cage in the following environment: 12 h light/dark regime, room temperature of 22 ± 1 °C, relative humidity of 55 ± 5%, and unlimited access to laboratory chow and tap water. The housing conditions and the laboratory food ingredients are described in detail in Potrebic et al. [23] and Perovic et al. [24], respectively. The experiments were performed in compliance with the guidelines in the directive on the protection of animals used for experimental and other scientific purposes (2010/63/EU).

### 2.2. EAE Induction and Evaluation

Experimental autoimmune encephalomyelitis was induced by active immunization using encephalitogenic emulsion, prepared and administered as previously described [5]. The animals were monitored daily for symptoms of EAE that were assessed based on the following scale: 0—unaffected, 1—tail atony, 2—hind limb weakness, 3—total hind limb paralysis, and 4—moribund state. Moribund state for two consecutive days was designated as the humane endpoint of the experiment. Both male and female rats were sacrificed at the onset (grade 1), peak (grade 3 or 4), or end (grade 0, after complete recovery). Each experiment used age- and sex-matched animals as naïve controls. Thus, all male and female experiments featured the following experimental groups: Control, Onset, Peak, and End.

The analyses of EAE parameters were defined as follows: day of onset—mean value of the first day any symptom was registered in each animal; duration of symptoms—mean value of the number of days during which each animal was graded with any grade higher than 0; duration of paralysis—mean value of the number of days during which each animal was graded with grade 3 or 4; maximal severity score—mean value of the maximal grade given to each animal during the disease; cumulative disease index—mean value of the sum of all grades given to each animal during the disease.

### 2.3. Blood and Tissue Collection

The animals were sacrificed between 9 and 10 am by asphyxia under gradually increasing CO_2_. Blood was collected by cardiac puncture to two separate collection tubes, one of which was coated with 0.4 M EDTA, to isolate the plasma. The other tube was uncoated and used to separate the serum. For plasma isolation, the tubes were centrifuged at 7500× *g* for 10 min at 4 °C. Plasma and serum samples were stored at −80 °C until further use.

After blood collection, the animals were perfused with cold saline, the hypothalamic tissue was dissected, and the adrenal glands and testes were isolated. Hypothalamus was dissected on ice as a block of tissue, using a coronal rat brain matrix, according to the following coordinates: rostral cut 2 mm anterior to the optic chiasm; caudal cut behind the mammillary bodies; lateral cuts 2 mm lateral to the 3rd ventricle; dorsal cut at the top of the 3rd ventricle. Hypothalamic tissue was stored in RNAlater^®^ RNA Stabilization Solution (Ambion^™^, Applied Biosystems by Thermo Fisher Scientific, Waltham, MA, USA) at −80 °C until further processing. The adrenal glands were either placed in RNAlater^®^ for RNA isolation or immediately frozen in liquid nitrogen and stored for protein isolation or steroid extraction. For these purposes, the adrenal glands were decapsulated. Testes were decapsulated and frozen in liquid nitrogen for steroid extraction. In one of the experiments, the adrenal glands were not decapsulated and were processed for histological evaluation.

### 2.4. Histological Staining and Volumetric Measurements

Adrenal glands were fixed in Bouin’s solution or in 4% neutral buffered formalin, dehydrated, embedded in paraffin, and cut on a microtome into 7 µm thick sections. Seventy µm apart sections were stained with hematoxylin and eosin. The micrographs were analyzed in Zeiss Zen software (Version 3.6). Each section’s total area and the medulla and cortex areas were measured, including zG, zF, and zR. The areas of zF and zR were analyzed as a whole (indicated as zF + zR) because of the difficulty in setting the boundary between them. The volumes of zG, ZF + zR, and medulla for each gland were derived from the values of surface areas and distance between sections.

### 2.5. Immunohistochemistry

The immunohistochemical labeling was performed on 7 µm thick sections of adrenal glands, prepared as described above. Three sections from three animals per group were stained as follows. After deparaffinization and rehydration, antigen retrieval was performed as follows: the slides were placed in 200 mL of 0.1 M citrate buffer (pH 6), heated in the microwave at 750 W for 5 min, and then left to cool at room temperature for 30 min. Following the endogenous peroxidase blocking, the slides were submerged in 0.1% TritonX-100 solution in 0.01 M phosphate-buffered saline (PBS) for 20 min at room temperature. To enhance the signal, Tyramide SuperBoost™ kit was used (Cat. No. B40943, Invitrogen™, Thermo Fisher Scientific, Waltham, MA, USA) and the manufacturer’s instructions were followed in detail. The blocking solution from the SuperBoost™ kit was used for the blocking step, after which the slides were incubated with the primary antibody (Ki-67, rabbit monoclonal, Vector Laboratories, Newark, CA, USA; Cat. No. VP-RM04; 1:100) at 4 °C, overnight. Poly-HRP-conjugated goat anti-rabbit secondary antibody, provided in the kit, was applied on the next day for 1 h at room temperature. Tyramide working solution, made as prescribed in the kit, was incubated for 10 min on the tissue slices. After the stop solution and washing in PBS, the slides were submerged into the solution of 4′,6-diamidino-2-phenylindole (DAPI) in PBS (0.1 μg/mL) for 20 min, to stain the nuclei. The slides were washed again 3× in PBS and mounted with Mowiol mounting medium (Calbiochem, Millipore, Darmstadt, Germany).

Immunofluorescent labeling was examined and photographed with Zeiss Axiovert microscope (Carl Zeiss GmbH, Vienna, Austria). The representative micrographs were arranged in Photoshop CC (Version 14.2, Adobe Systems Inc., San Jose, CA, USA). The staining and counting were performed in three sections per adrenal gland (280 µm apart, through the mid portion of the gland), in three animals per group. The micrographs were analyzed in Zeiss Zen software (Version 3.6) and the results are expressed as the number of Ki-67 positive nuclei per mm^2^ of the zG which serves as a stem cell pool of the adrenal gland [25].

### 2.6. Steroid Extraction

Decapsulated adrenal glands and testicles (*n* = 6/group) were placed into PBS (5 μL of PBS per 1 mg of tissue) and homogenized individually. Further steroid isolation procedure was performed as described previously in detail [7]. Steroid content was diluted in PBS with 0.1% bovine serum albumin (BSA; Sigma, St. Louis, MO, USA) and used for hormone measurements.

### 2.7. Hormone Measurements

The levels of ACTH in plasma samples were measured using an ACTH EIA Kit (Phoenix Pharmaceuticals, Inc., Burlingame, CA, USA; Cat. No. EK-001-21). Progesterone and corticosterone levels in sera and tissue steroid isolates were measured using the kits by Cayman Chemical Company (Ann Arbor, MI, USA; Cat. No. 582601) and Enzo (Farmingdale, NY, USA; Cat. No. ADI-900-097), respectively. The manufacturers’ instruction sheets were followed in detail for all ELISA measurements. After method optimization, plasma/serum samples and adrenal and testicular tissue steroid isolates were adequately diluted. The results for plasma/serum samples are presented per mL of plasma/serum, while steroid hormone levels for tissue steroid isolates are expressed per g of the corresponding tissue.

### 2.8. RNA Isolation and qPCR Analysis

RNA was isolated using the RNeasy Mini Kit (QIAGEN, Hilden, Germany) according to the manufacturer’s instructions. Reverse transcription and the analysis of the acquired cDNA samples by quantitative PCR (qPCR) were performed as previously described in our experiments [5]. For qPCR, performed in QuantStudio™ 3 Real-Time PCR System, TaqMan^®^ and SYBR^™^ green chemistry were used (all by Applied Biosystems by Thermo Fisher Scientific, Waltham, MA, USA). Primer pairs used for SYBR^™^ green chemistry are shown in Table 1. The TaqMan^®^ probes used were *Cyp11b1* (Rn02607234_g1) and *Gapdh* (Rn01462661_g1). The expression levels of target genes were quantified by the comparative 2^−∆Ct^ method, using *Gapdh* as a reference gene. The reference gene for hypothalamic tissue was chosen based on our previous study [5]. For adrenal tissue, *Gapdh* was identified as the optimal reference gene among *Actb*, *B2m*, *Gapdh*, *Hprt*, *Ppia*, and *Rn18s*, analyzed using RefFinder [26].

### 2.9. Protein Isolation and Western Blot

In each experiment, six decapsulated adrenals were pooled for protein isolation for each experimental group. The proteins were isolated with RIPA buffer (R0287, Sigma, St. Louis, MO, USA) enriched with protease and phosphatase inhibitors (Cat. No. 88668, Thermo Fisher, Rockford, IL, USA). The samples were loaded (10 µg of proteins per well) onto 10% SDS polyacrylamide gels and separated via gel electrophoresis. For the analysis of 3β-HSD, the staining of total proteins was performed prior to the blocking step, using MemCode reversible protein stain kit for PVDF membranes (Cat. No. 24585, Thermo Fisher Scientific, Waltham, MA, USA), and following the manufacturer’s instructions in detail. Subsequent steps of Western blot analysis were performed as previously described in detail [6,7]. The list of antibodies used is given in Table 2. The blot images were subjected to densitometric analysis via the ImageJ software package (version 2.1.0/1.53c; Java 1.8.0_66). The analysis was performed by measuring the optical density (OD) of protein bands of interest in each lane and normalizing them to the OD of either β-actin (for StAR and P450scc analyses) or total protein stain (for 3β-HSD analyses). The normalized ODs from each group are presented as mean fold change (±SEM) relative to the Control, which was set as 1 ± SEM. Original figures can be found in Supplementary Materials. 

### 2.10. Statistical Analysis and Data Presentation

Statistical analyses were performed in GraphPad Prism 8 (version 8.0.2 for Windows, GraphPad Software, La Jolla, CA, USA). Since the data did not fulfil both of the criteria of normality and homoscedasticity for parametric tests, i.e., the criterium of homogeneity of variance was not met, the non-parametric Kruskal–Wallis test was applied to analyze differences between multiple groups followed by uncorrected Dunn’s post hoc test, to compare the differences between Control, Onset, Peak, and End groups within the animals of each sex, as well as differences between the corresponding groups of males and females (e.g., Control male vs. Control female). Mann–Whitney test was used to examine the differences between the two groups of animals. The results are presented as mean ± SEM for each group of animals. Statistical significance in the graphs indicates the difference between each of the EAE groups compared to the corresponding Control group within each sex, and is labeled as: * *p* < 0.05, ** *p* < 0.01; or between males and females from the corresponding EAE groups of animals, and is labeled as: # *p* < 0.05, ## *p* < 0.01. The graphs were made in GraphPad Prism 8.

## 3. Results

### 3.1. Characterization of EAE Course and Parameters in Male and Female DA Rats

**Figure A1:**
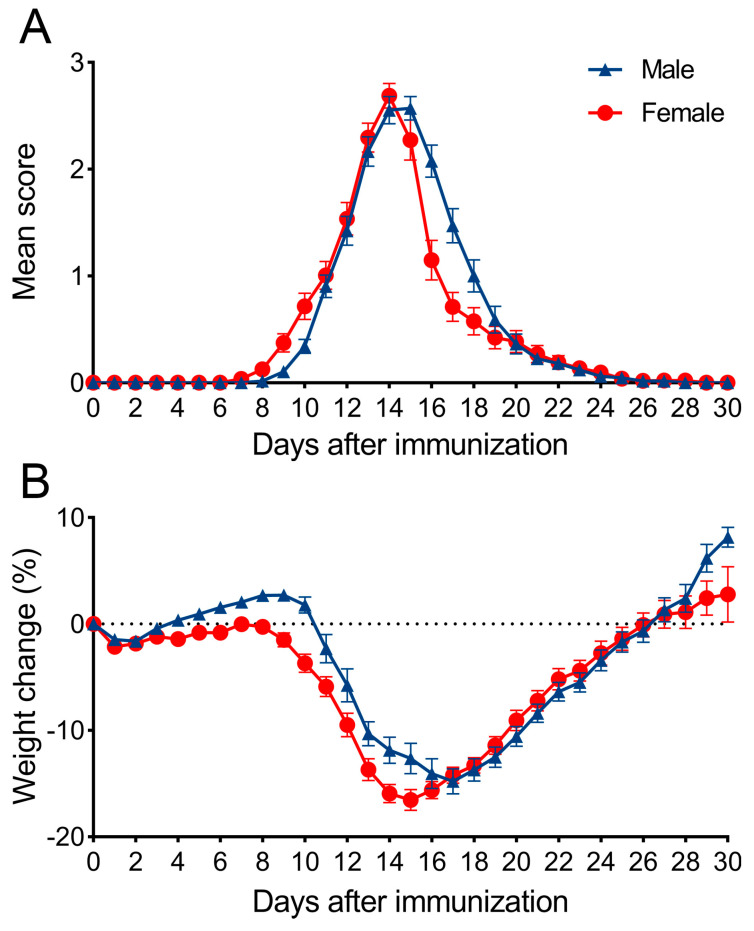
EAE course and body weight change in male and female DA rats. The symptoms of EAE and body weight were monitored daily for 30 dpi in males (blue triangles) and females (red circles). (**A**) Mean neurological score ± SEM; (**B**) mean change in body weight compared to the day of immunization (% ± SEM). The figure is presented as cumulative data from 5 experiments in both sexes (*n* = 96 for males, *n* = 84 for females).

The active immunization resulted in a monophasic course of EAE, as already described in our studies in DA rats of both sexes [5,6,7]. The course of EAE for males and females and the accompanying weight change are shown in Appendix A, Figure A1A and Figure A1B, respectively. The first symptoms were noted 8 days post immunization (dpi) in males and 7 dpi in females; the peak of the disease in males was registered on two consecutive days—14 dpi (mean score 2.50 ± 0.14) and 15 dpi (mean score 2.50 ± 0.13), and on 14 dpi in females (mean score 2.55 ± 0.13), while no symptoms were registered after 27 dpi in animals of both sexes Figure A1A). The course of EAE was followed by a decrease in body weight in both sexes, reaching a nadir around the time of disease peak −14.79 ± 1.18% on 17 dpi in males and −16.55 ± 0.98% on 15 dpi in females; Figure A1B).

The analysis of the parameters of EAE based on 5 male and female experiments performed in our laboratory is shown in Table 3. The first manifestations of the disease are registered earlier in females than in males (*p* = 0.047). While there was no statistical difference in the duration of paralysis or cumulative disease severity, males had significantly higher maximal severity score (*p* = 0.021).

### 3.2. Adrenal Weight and Volumetric Measures in Male and Female Rats during EAE

Adrenal weight measurement and volumetric analyses were performed in the Control, Onset, Peak, and End groups of animals of both sexes. The absolute values with the comparison between sexes are presented in Table 4. As expected, adrenal weight was lower in males than in females in Control rats and during the disease (*p* < 0.01 in all compared groups). Adrenal volume was significantly lower in Control males compared to Control females (*p* = 0.014) as well as at the end of EAE (*p* = 0.033). Similarly, cortex volume was significantly smaller in males than females in Control (*p* = 0.014) and End groups (*p* = 0.037). The volume of zG was different between sexes, i.e., lower in control males (*p* = 0.025), while the volume of zF + zR was lower in males than females of Control (*p* = 0.017), Onset (*p* = 0.035), and End (*p* = 0.046) groups of rats.

The analyses of these parameters throughout the disease, within the animals of each sex, have shown that the adrenal weight was significantly increased at the onset (~15%, *p =* 0.0082) and peak of EAE in males (~30%, *p <* 0.0001), and only at the peak of EAE in females (~17%, *p =* 0.0061). Based on volumetric measurements, the differences were observed only in males in the Peak group compared to the Control group. Namely, the following parameters were significantly increased: the volume of the whole adrenal glands (~36%, *p* = 0.023), the volume of adrenal cortex (~35%, *p* = 0.024), and the volume of zF + zR of the adrenal cortex (~37%, *p* = 0.024). The volume of adrenal medulla has shown a trend of increase (~53%, *p* = 0.056). None of the volumetric parameters were significantly changed during EAE in female rats.

**Figure 1:**
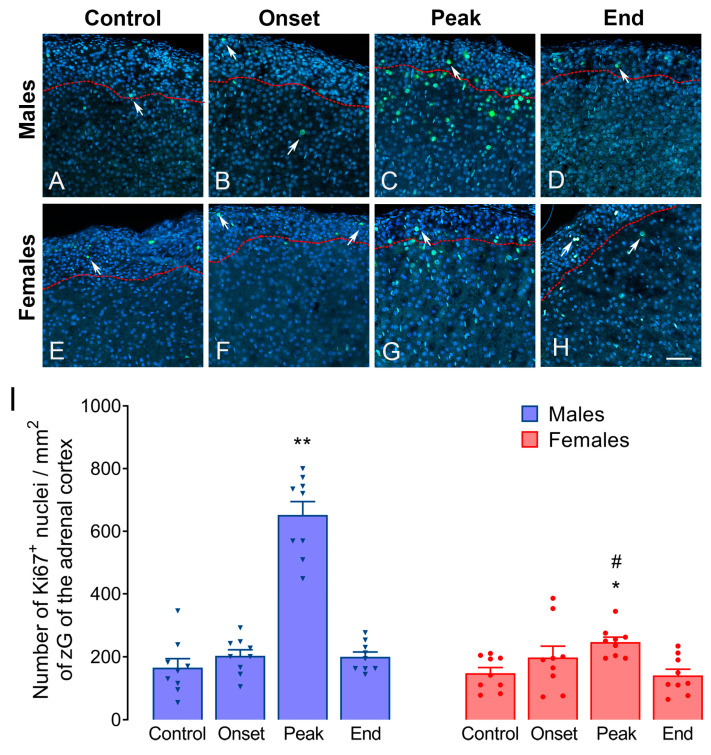
Ki-67 labeling in the adrenal glands. The adrenal glands were fixed in neutral buffered formalin and embedded in paraffin. Seven µm thick sections were labeled using Ki-67 antibody (green) and counterstained with DAPI (blue). Top row (**A**–**D**) represents sections from male and bottom row (**E**–**H**) from female adrenal glands: (**A**,**E**) Control, (**B**,**F**) Onset, (**C**,**G**) Peak, (**D**,**H**) End. Arrows indicate Ki-67-positive nuclei. The red lines indicate the border of zG. Scale bar: 50 µm, applies to all. Quantification results are presented in (**I**). The asterisks indicate the differences compared to Control within the animals of the same sex. * *p* < 0.05, ** *p* < 0.01; octothorpes indicate the differences between the corresponding groups of males and females; # *p* < 0.05 (*n* = 3 sections per adrenal gland, from *n* = 3 animals per group). Kruskal–Wallis test followed by uncorrected Dunn’s post hoc test.

To elucidate the cause of the more pronounced differences in the adrenal volumetric parameters in male rats, the adrenal glands of rats of both sexes were labeled for Ki-67, the marker of proliferation (Figure 1).

A few Ki-67-positive nuclei could be observed in the adrenal cortex of male rats from the Control group (165.6 ± 34.00 Ki-67-positive nuclei per mm^2^ of zG; Figure 1A). A similar staining was noted in the Onset group (202.5 ± 29.47 Ki-67-positive nuclei per mm^2^ of zG; Figure 1B), while an increase in the number of Ki-67-positive cells in this cortical region was observed in the Peak group of male rats (653.2 ± 47.54 Ki-67-positive nuclei per mm^2^ of zG, *p* = 0.0001; Figure 1C). The staining comparable to the one in Control was observed at the end of EAE in male rats (201.4 ± 18.60 Ki-67-positive nuclei per mm^2^ of zG; Figure 1D). In females, Ki-67 labeling was similar as in males, in the Control group (147.4 ± 23.59 Ki-67-positive nuclei per mm^2^ of zG; Figure 1E), as well as in the Onset group (198.0 ± 46.26 Ki-67-positive nuclei per mm^2^ of zG; Figure 1F). In the Peak group of females, a significant increase in Ki-67 positive nuclei was observed (246.6 ± 15.82 Ki-67-positive nuclei per mm^2^ of zG; *p* = 0.034; Figure 1G), but this increase was significantly less pronounced than in males (*p* = 0.049). At the end of the disease in female rats, the staining was comparable to the Control group (139.6 ± 24.37 Ki-67-positive nuclei per mm^2^ of zG; Figure 1H). Quantification results are presented in Figure 1I.

### 3.3. Crh Expression in the Hypothalamus and Circulating ACTH Levels during EAE

**Figure 2:**
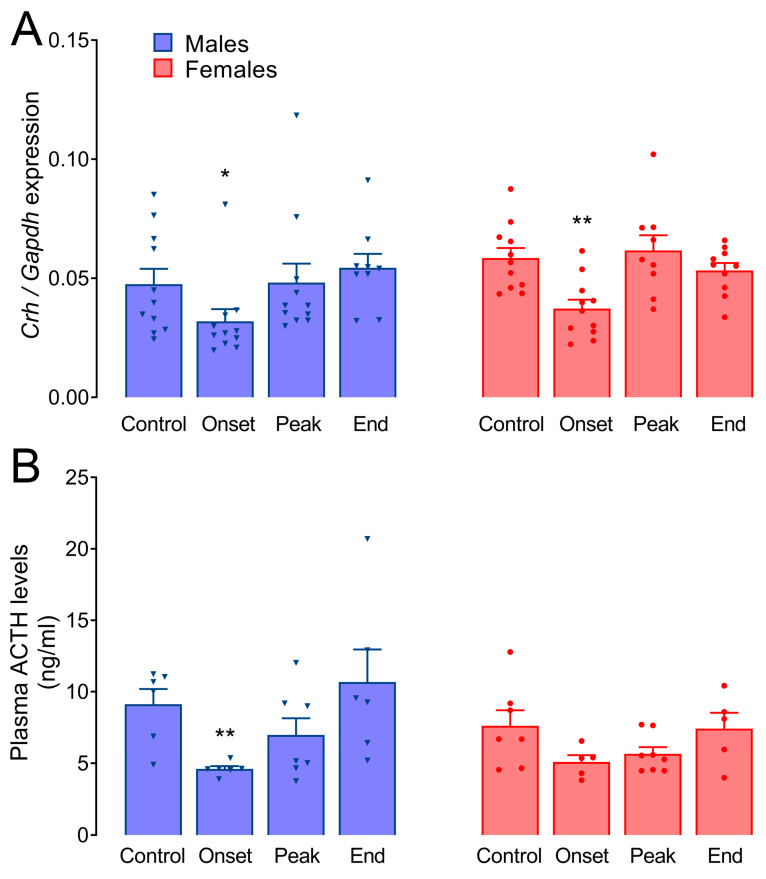
Hypothalamic *Crh* expression and circulating ACTH levels. (**A**) *Crh* expression was analyzed via qPCR in the hypothalamic tissue of the rats of both sexes. The results are presented as mean expression (relative to *Gapdh*) ± SEM. (**B**) Plasma ACTH levels were measured using the ELISA method. The results are presented as mean ACTH levels ± SEM. The asterisks indicate the differences compared to Control within the animals of the same sex. * *p* < 0.05, ** *p* < 0.01; Kruskal–Wallis test followed by uncorrected Dunn’s post hoc test.

**Figure A2:**
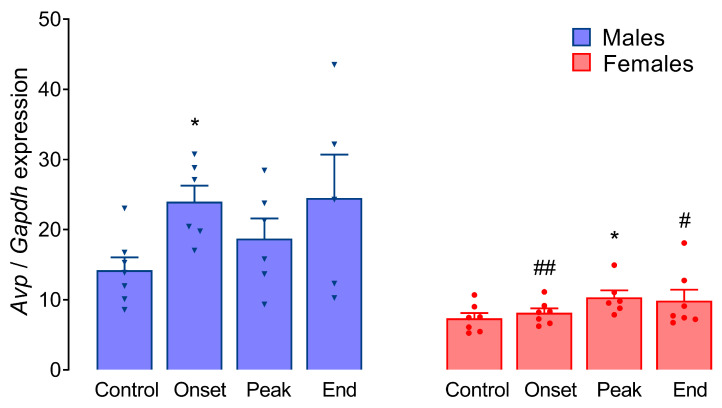
The expression of *Avp* in the hypothalamic tissue of male and female DA rats during EAE. The results are presented as mean expression (relative to *Gapdh*) ± SEM, from a representative experiment. The asterisks indicate the differences compared to Control within the animals of the same sex. * *p* < 0.05; octothorpes indicate the differences between the corresponding groups of males and females; # *p* < 0.05, ## *p* < 0.01. Kruskal–Wallis test followed by uncorrected Dunn’s post hoc test.

The expression of *Crh* in the hypothalamus was downregulated only at the onset of EAE, about 35% in males (*p* = 0.025) and females (*p* = 0.0038; Figure 2A). In a representative experiment, the expression of *Avp* in the hypothalamus was upregulated at the onset of EAE in males (*p* = 0.035) and at the peak in females (*p* = 0.029; Figure A2). A significantly lower expression of *Avp*, compared to the corresponding male groups, was observed in females at the onset (*p* = 0.0006) and end of EAE (*p* = 0.0011). Mean relative expression values ± SEM for these and all subsequent gene expression analyses are presented in Table S1.

Basal ACTH levels were similar in males (9.12 ± 1.07 ng/mL) and females (7.59 ± 0.83 ng/mL; Figure 2B). A significant decrease in ACTH levels was registered only at the onset of EAE in male rats (4.60 ± 0.23 ng/mL, *p =* 0.0024), while in females, it was decreased (5.09 ± 0.48 ng/mL), but not significantly (*p =* 0.068), compared to the respective Control groups.

### 3.4. Corticosterone Levels in Males and Females during EAE

**Figure 3:**
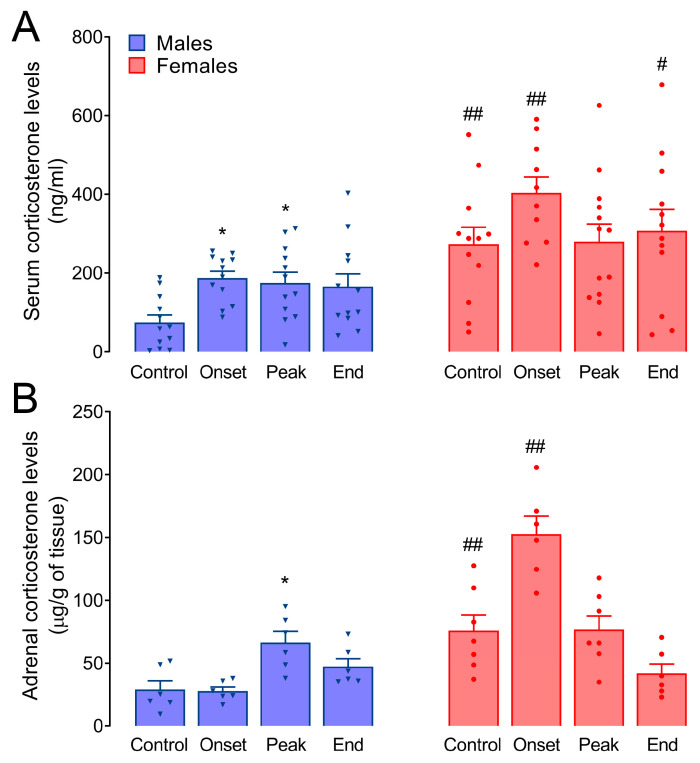
Serum and adrenal corticosterone levels. ELISA was used to measure corticosterone levels in (**A**) serum (ng/mL) and in (**B**) steroid extracts from the adrenal tissue (µg/g of tissue). The results are presented as mean corticosterone levels ± SEM for each group of animals. Asterisks indicate the differences compared to Control within the animals of the same sex. * *p* < 0.05,; octothorpes indicate the differences between the corresponding groups of males and females; # *p* < 0.05, ## *p* < 0.01. Kruskal–Wallis test followed by uncorrected Dunn’s post hoc test.

Basal serum corticosterone levels were lower in males (74.61 ± 19.03 ng/mL) than in females (261.5 ± 42.32 ng/mL; *p* = 0.0007; Figure 3A). The course of EAE affected serum corticosterone levels in males throughout the disease, as they were significantly elevated at the onset (187.40 ± 17.33 ng/mL, *p* = 0.018) and peak (175.30 ± 26.98 ng/mL, *p* = 0.039), while an increasing trend was observed at the end of EAE (165.70 ± 32.47 ng/mL); however, statistical significance was not reached (*p* = 0.081). No significant differences in serum corticosterone levels were observed in females during EAE. Nevertheless, a trend toward an increase was noted at the onset of the disease (405.40 ± 46.38, *p* = 0.059). Consequently, female corticosterone levels remained significantly higher than in males at the onset (*p* = 0.0083) and end of EAE (*p* = 0.046), but not at the peak of the disease.

Similarly, in adrenal steroid extracts, basal corticosterone levels were lower in males (29.00 ± 7.05 µg/g of tissue) than in females (75.86 ± 12.49 µg/g of tissue, *p* = 0.0072). In males, adrenal corticosterone levels were significantly increased at the peak of EAE (66.48 ± 10.76 µg/g of tissue, *p* = 0.028). In contrast, no significant increase in females compared to the corresponding Control group was recorded, but a trend toward an increase was observed at the onset of the disease (152.80 ± 16.68 µg/g of tissue, *p* = 0.11; Figure 3B). This is followed by significant differences between female and male adrenal corticosterone levels which remain higher in females at the onset of EAE (*p* < 0.0001). However, this difference was not observed at the peak of the disease.

### 3.5. Progesterone Levels in Serum, Adrenals, and Testes of Male Rats during EAE

**Figure 4:**
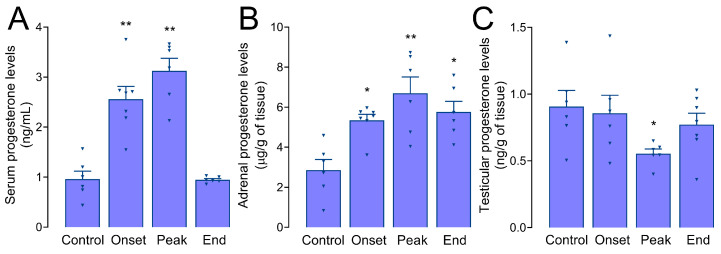
Progesterone levels in the serum, adrenals, and testes of male rats during EAE. Progesterone levels were measured using ELISA in males in (**A**) serum (ng/mL), (**B**) adrenal steroid extracts (µg/g tissue), and (**C**) testes (ng/g tissue). The results are presented as mean progesterone concentration ± SEM for each group of animals. * *p* < 0.05, ** *p* < 0.01; Kruskal–Wallis test followed by uncorrected Dunn’s post hoc test.

We found significantly elevated progesterone levels in serum samples of males at the onset (2.57 ± 0.27 ng/mL, *p =* 0.008) and the peak of EAE (3.13 ± 0.28 ng/mL, *p =* 0.003) compared to control levels (0.95 ± 0.17 ng/mL) (Figure 4A). Next, we assessed progesterone levels in adrenal steroid extracts of male rats during EAE (Figure 4B). The results have shown that, compared to control levels (2.85 ± 0.58 µg/g of tissue), progesterone levels were significantly increased at the onset (5.34 ± 0.30 µg/g of tissue, *p =* 0.028), peak (6.70 ± 0.88 µg/g of tissue, *p =* 0.002) and end of the disease (5.76 ± 0.53 µg/g of tissue, *p =* 0.014). Also, to further explore the origin of serum progesterone in males during EAE, progesterone levels were measured in testicular tissue steroid extracts (Figure 4C). While no difference was found at the onset and end of EAE, progesterone levels were significantly decreased at the peak of the disease (0.55 ± 0.036 ng/g of tissue, *p =* 0.034) compared to control levels (0.91 ± 0.13 ng/g of tissue).

**Figure A3:**
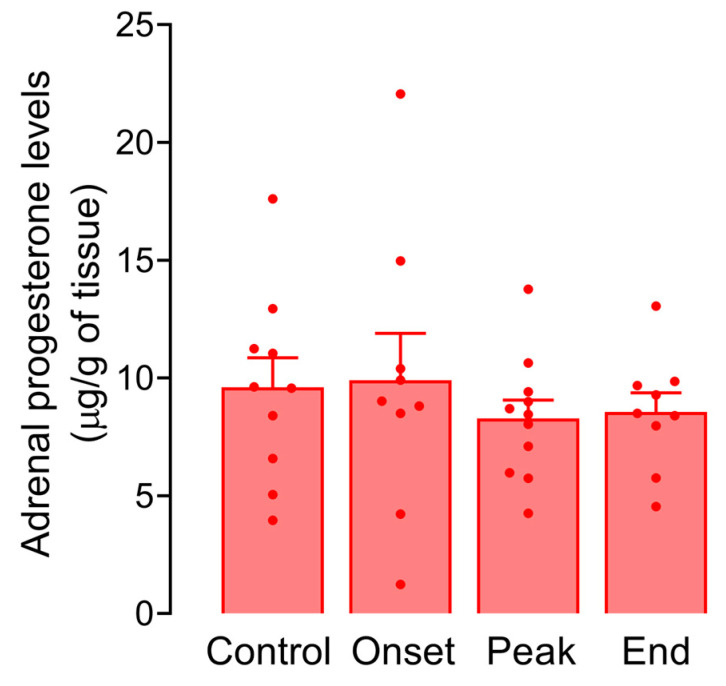
Progesterone levels in the adrenal glands of female rats during EAE. The results are presented as mean progesterone concentration (in µg/g tissue) ± SEM. Kruskal–Wallis test followed by uncorrected Dunn’s post hoc test.

On the other hand, we have previously registered an increase in serum and ovarian progesterone levels in female rats during EAE [5,7]. Despite the increasing trend in adrenal corticosterone levels at the onset, no differences were found in adrenal progesterone levels between the Control female group and any female groups with EAE (Figure A3).

### 3.6. Gene Expression of Adrenal Steroidogenic Pathway Components during EAE

**Figure 5:**
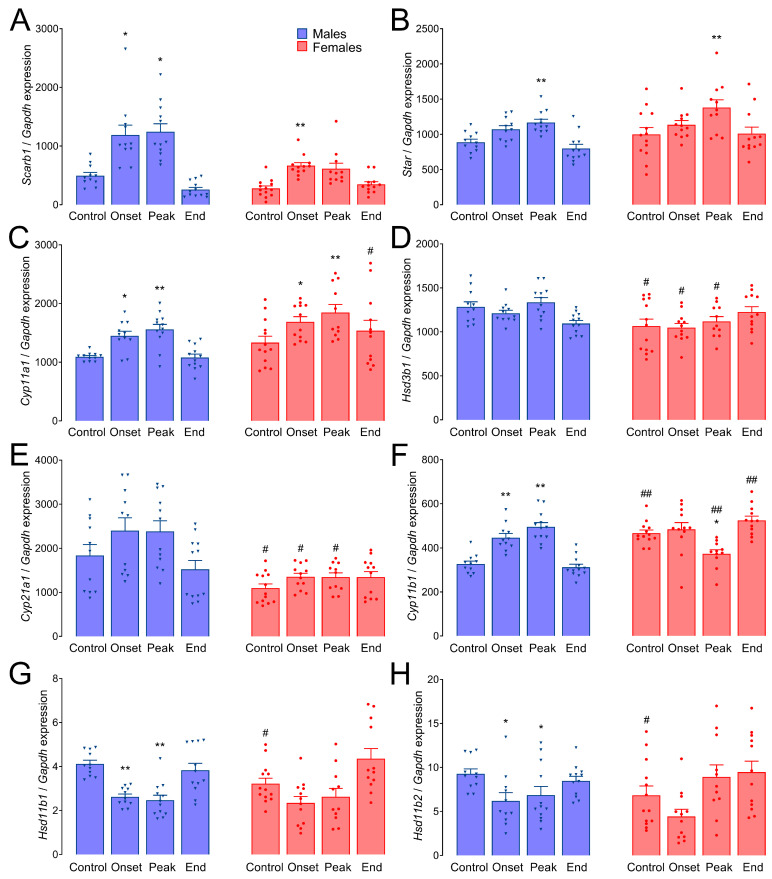
Gene expression of adrenal steroidogenic pathway components in males and females. The expression of genes involved in the steroidogenic pathway in the adrenal glands was analyzed by qPCR using SYBR green or TaqMan methodology. (**A**) *Scarb1*; (**B**) *Star;* (**C**) *Cyp11a1*; (**D**) *Hsd3b1*; (**E**) *Cyp21a1*; (**F**) *Cyp11b1*; (**G**) *Hsd11b1*; (**H**) *Hsd11b2*. The results are presented as mean expression (relative to *Gapdh*) ± SEM. Asterisks indicate the differences compared to Control within the animals of the same sex, * *p* < 0.05, ** *p* < 0.01; octothorpes indicate the differences between the corresponding groups of males and females; # *p* < 0.05, ## *p* < 0.01. Kruskal–Wallis test followed by uncorrected Dunn’s post hoc test.

The expression of *Scarb1* in adrenal glands was upregulated about two-fold during the onset and peak of EAE in male rats, and only at the onset in female rats (Figure 5A). Messenger RNA levels of *Star* were significantly increased only at the peak of the disease in the adrenals of rats of both sexes (Figure 5B). The expression of *Cyp11a1* was upregulated during the symptomatic phases of EAE in both males and females (Figure 5C). No significant differences were registered in the expression of either *Hsd3b1* (Figure 5D) or *Cyp21a1* (Figure 5E). However, these two genes were both significantly less expressed in the Control, Onset, and Peak groups of female rats, compared to the corresponding male groups. The levels of *Cyp11b1* mRNA were affected differently depending on the sex—they were significantly elevated at the onset and peak in males, while in females, they were significantly decreased at the peak of EAE. Moreover, the expression of *Cyp11b1* was significantly higher in the Control and End groups of females, compared to the corresponding male groups, while it was significantly lower at the peak of EAE (Figure 5F). The expression of *Hsd11b1* and *Hsd11b2* was affected similarly—these genes were downregulated at the onset and peak of EAE in males, while they were unchanged in females, and both their basal levels were significantly lower in females than in males (Figure 5G,H).

### 3.7. Protein Levels of StAR, P450scc, and 3β-HSD in Adrenal Glands

**Figure 6:**
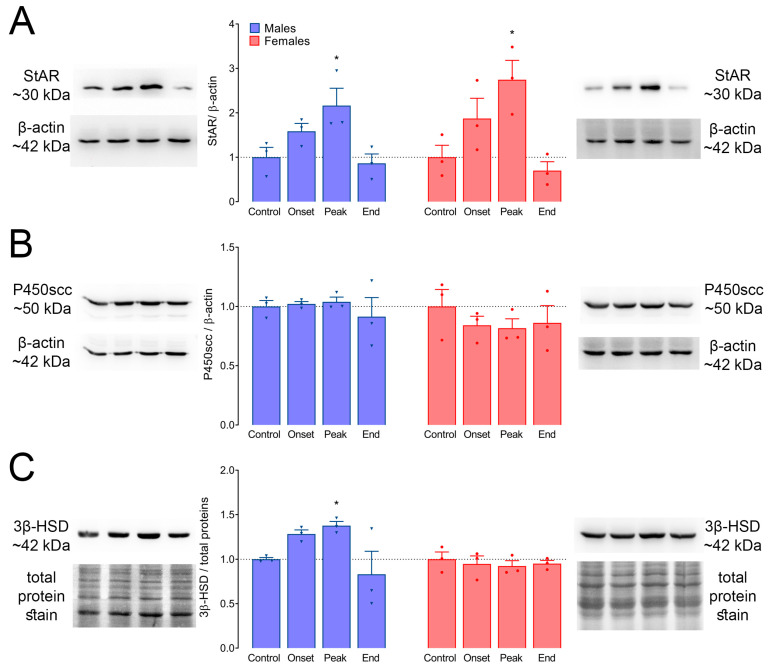
Protein levels of StAR, P450scc, and 3β-HSD in the adrenal glands of rats during EAE. The levels of proteins involved in steroidogenesis in the adrenal glands were analyzed using Western blot. (**A**) StAR, (**B**) P450scc, and (**C**) 3β-HSD. The results were calculated as the optical density of the target proteins relative to β-actin (StAR and P450scc) or total proteins (3β-HSD), normalized to Control (presented as mean ± SEM), and plotted as mean fold change ± SEM (from three independent Western blot experiments). Representative blot images for each protein of interest and its loading control are presented on the left for males and on the right for females. The order of the bands in each blot corresponds to the order of the groups on the graphs (from left to right: Control, Onset, Peak, End). Unprocessed blot images are provided in the Figure S1. * *p* < 0.05; Kruskal–Wallis test followed by uncorrected Dunn’s post hoc test.

Next, we analyzed the profiles of StAR, P450scc, and 3β-HSD expression at the protein level (Figure 6). StAR protein levels were significantly increased at the peak of EAE in males, about 2× compared to Control levels (*p =* 0.042; Figure 6A, left), and in females, this increase was about 3× compared to Control (*p =* 0.032; Figure 6A, right). The levels of P450scc remained unchanged, both in males (Figure 6B, left) and in females (Figure 6B, right). Western blot analyses have shown significantly greater levels of 3β-HSD protein at the peak of EAE in males (~40% increase, *p =* 0.031; Figure 6C, left), while no differences in its levels were observed in females (Figure 6C, right).

## 4. Discussion

Numerous studies have shown that MS triggers activation of the HPA axis [10]. However, the significance of this phenomenon for the onset and/or progression of the disease still needs to be clarified. Meanwhile, glucocorticoids remain the drug of choice in the treatment of acute relapses of MS, as they prevent the nerve and tissue damage associated with inflammation. The exact mechanisms of action of glucocorticoids are still relatively unknown. Most of the studies in MS and in animal models of this disease have not included sex as a variable, although there are sex-specific differences in HPA axis function.

In response to physical and psychological stressors, female rats secrete higher absolute corticosterone concentrations than males [28]. Females have larger adrenal glands, which seem highly dependent on circulating gonadal steroid hormones [29]. In contrast to rats, men have been found to have slightly higher basal cortisol levels than women [30], but both women and female rats show a more robust HPA response to various stressors [29,31]. However, our results suggest that more pronounced changes in adrenal function occur in male rats during EAE.

Here, we show that the sex-specific response of the HPA axis manifests at the level of the adrenal glands in acute monophasic EAE. Adrenal activity is increased during EAE in both males and females, as evidenced by enlarged adrenal glands, increased StAR gene and protein expression, and increased adrenal corticosterone levels in males. This is reflected in the elevation of serum corticosterone levels in males but not females. Elevated plasma cortisol levels have also been found in MS patients, although these individuals showed normal responses to ovine ACTH and CRH [32]. On the other hand, diurnal patterns of cortisol secretion and HPA feedback regulation were unaffected in different subgroups of MS patients [33]. However, it has also been postulated that MS patients exhibit impaired glucocorticoid-mediated feedback [34,35,36]. In general, high levels of cortisol in the CSF have been associated with slower disease progression, particularly in females with secondary progressive MS [37]. Importantly, sex-specific differences have not been explored in these studies. Interestingly, cortisol levels are lower during the luteal phase of the menstrual cycle [38], coinciding with premenstrual worsening of MS symptoms [39].

An increase in corticosterone levels was previously found in both male [40] and female DA rats with EAE [8,41]. It is important to note that the HPA axis response in DA rats varies depending on the immunogen used for EAE induction—MOG elicits a more robust response than MBP [41]. When using a spinal cord homogenate for immunization, we here found no significant changes in serum corticosterone levels during EAE in female animals, a departure from our previous findings [8]. However, we here also show that the upregulation of *Cyp11b1* expression, the enzyme converting DOC to corticosterone, occurs during EAE in male adrenals only. Moreover, the variability in serum corticosterone measurements could potentially be attributed to the higher rate of metabolic clearance of corticosteroids in female rats compared to males [42]. Interestingly, the mRNA levels of *Hsd11b1* and *Hsd11b2*, which produce the active glucocorticoid and the inactive 11-keto metabolites, respectively, are only inhibited in males during the disease. Little can be concluded on physiological significance of these data without more detailed experiments, but it is of note that knocking out either *Hsd11b1* or *Hsd11b2* results in enlarged adrenal glands in rodents [43,44].

Our results show that adrenal gland weight increases during EAE in both males and females. However, we observed a more pronounced weight increase in males, and further analyses confirmed a significant increase in the volumes of adrenal cortex in males only. Males also show higher proliferation rate in zG at the peak of the disease, based on Ki-67 staining. As Ki67-positive cells differentiate, they migrate centripetally [25], but we cannot claim that they will differentiate into steroid-producing cells. However, this provides at least partial explanation for the observed enlargement of adrenal glands. Importantly, postmortem analysis in MS patients revealed enlarged adrenal glands [45].

Our previously published data show a higher ACTH content in the pituitary gland and a larger volume of corticotropes in females at the peak of the disease [8]. Here, we found consistently lower plasma ACTH levels at the onset of EAE in male rats, suggesting that the release of ACTH is inhibited at the level of the pituitary gland, in line with the decreased hypothalamic *Crh* levels. No difference in plasma ACTH levels was observed in Lewis rats with EAE [46,47], while one study reported a significant increase in the same animal model [48]. These discrepancies are difficult to explain as the disturbed homeostasis in this animal model is due to more than one stressor. The number of CRH neurons in the paraventricular nucleus of MS patients increases [49,50], in contrast to a decrease in the mRNA levels of CRH in the hypothalamus during disease onset presented here. Nevertheless, *Crh* downregulation appears to be a common feature in several immune-mediated disease models, including EAE [51], and this phenomenon precedes the worsening of symptoms [52]. The inhibition at the hypothalamic and pituitary levels observed here in males could result from glucocorticoid feedback [53]. On the other hand, in addition to CRH, AVP can stimulate ACTH production, and its mRNA and protein levels are elevated in chronic inflammatory stress models [51], which we have confirmed here. Unsurprisingly, the AVP gene expression is stimulated during EAE, considering that animals with EAE show diminished water intake during the symptomatic phase of the disease [54]. Increased plasma osmolality is a physiological condition that has been shown to inhibit hypothalamic CRH mRNA levels and secretion, as well as basal and stress-stimulated pituitary ACTH secretion, and has opposite effects on the secretion of ACTH and AVP from the adenohypophysis and neurohypophysis, respectively [55,56,57].

Gonadal hormones are partially responsible for the sex-specific differences in the glucocorticoid response to stress. In rats, castration leads to a significant increase in basal and stress-induced corticosterone secretion, whereas ovariectomy decreases basal corticosterone levels and its pulsatility, as well as its secretion in response to stress [29]. Generally, androgens decrease the activity of the HPA axis, while estrogens stimulate it [58]. Considering that serum testosterone levels are significantly reduced in males while estradiol levels are unchanged in females in our EAE model [5], we can conclude that such a status of sex hormones might contribute to the sex differences in the amplitude of the adrenal response to disease. On the other hand, activation of the HPA axis, especially in cases of repetitive or chronic stress, such as during EAE, has an inhibitory effect on estrogen and testosterone secretion. Given the results presented here and our previously published work, we cannot exclude the possibility that, in addition to inflammation in the hypothalamus, an increase in serum corticosterone levels in male rats also contributes to the previously reported suppression of the HPG axis [5,6]. Although LH levels are decreased during EAE in rats of both sexes, this decrease is more prominent, i.e., long-lasting in males than females, together with the downregulation of hypothalamic *Gnrh1* expression in males only [5]. This could be a result of a higher amplitude of adrenal response in the form of corticosterone production in males compared to females, as shown here, because chronic exposure to glucocorticoids decreases hypothalamic GnRH mRNA levels in rats [59], suggesting different mechanisms of modulation of the HPG axis during EAE between the sexes.

Also, there is a sex difference in adrenal response to the disease, mirrored in increased production of progesterone, a precursor of corticosterone, in male adrenal glands only. This aligns with the induction of 3β-HSD protein levels in male, but not female, adrenal glands. It is difficult to explain the discrepancy between mRNA and protein levels for 3β-HSD observed in male rats, but it could be attributed to post-transcriptional, translational, and/or post-translational regulation. Similarly to the results presented here, a significant discrepancy between serum concentrations of progesterone and corticosterone was previously reported during restraint stress in rats of both sexes [60]. The increase in serum progesterone levels occurs in both sexes during EAE; interestingly, in females, this increase originates from the ovaries [7], while in males, this progesterone seems to be produced by the adrenals. Even though adrenal corticosterone levels showed an increasing trend at the onset of EAE in females, this was not coupled with alterations in adrenal progesterone levels. Overall, these results imply that the regulation of metabolism and secretion of steroid hormones in the adrenal glands during EAE is complex and multifaceted. Progesterone, classically considered a female sex hormone, has been associated with female preponderance in various autoimmune diseases, while in MS patients, high progesterone levels during pregnancy have been associated with a lower frequency of relapses [61]. In the context of this study, it is essential to point out that plasma progesterone levels are significantly higher in male MS patients than in healthy men [62], suggesting that the increase in progesterone may be one of the protective mechanisms in response to MS/EAE since progesterone and synthetic progestins have immunomodulatory and anti-inflammatory properties mediated by binding to progesterone or glucocorticoid receptors [63]. Therefore, the use of progestins in men in inflammatory conditions should be explored in greater depth.

As previously mentioned, despite the lower prevalence, men are more prone to having a worse MS course than women [2]. Although we previously reported no difference in the EAE severity between males and females [5], we here stand corrected because the detailed analysis of disease parameters across experiments showed that the maximal severity score is slightly but significantly higher compared to female rats. This is consistent with the literature showing that male rats develop more severe acute monophasic EAE than females and that spinal cord microglia/macrophages are more activated and produce more pro-inflammatory cytokines that favor the differentiation of highly pathogenic IL-17 + IFN-γ + cells in males [64]. Glucocorticoids, generally known for their anti-inflammatory and protective properties, can paradoxically have pro-inflammatory effects depending on the time, concentration, and duration of exposure, particularly in the CNS [65]. Thus, it is possible that a higher adrenal response, resulting in an increase in serum corticosterone levels that persists during the development and resolution of EAE in male rats, could have both a pro-inflammatory effect that contributes to the worsening of the disease’s severity and a protective anti-inflammatory action required for the spontaneous recovery. However, further experiments are needed to verify this.

The observed sex differences in the adrenal activation during EAE could be attributed to higher baseline levels of corticosterone in female rats, suggesting that, although the female sex is at a higher risk for autoimmune diseases, it may be better equipped to respond to inflammatory challenges. Our results in males show that the role of glucocorticoids is more complex than the traditional anti-inflammatory/protective point of view—since the elevation in corticosterone levels persists throughout the disease in male rats, it may, except for presenting an internal anti-inflammatory mechanism, also contribute to disease pathology. Therefore, it is crucial to consider the potential risks and benefits of administering glucocorticoids for CNS inflammation, especially in male patients. While they may alleviate symptoms, they may also exacerbate clinical outcomes [66]. Our findings indicate that males and females increase their circulating progesterone levels during EAE via different sources. The use of various mechanisms for the same outcome in both sexes suggests that progesterone may play an essential role in regulating the disease.

## 5. Conclusions

This study shows that acute EAE leads to a more robust adrenal gland activation in male rats of *Dark Agouti* strain, together with a higher severity of the disease. The more pronounced adrenal response in males is reflected in a more prominent enlargement of adrenal compartments, a persistent increase in serum corticosterone throughout the disease, and in elevated adrenal corticosterone and progesterone. This detailed analysis of the events unfolding at the level of adrenal glands during EAE deepens the knowledge of the role of glucocorticoids during immune challenges. The results presented here add to our previous work on the disruption of HPG axis functions at all levels during EAE in both sexes. Taken together, our research strengthens the perception of the complex interactions between HPG and HPA axes, and highlights the importance of a systematic approach in the research of sex differences.

## Figures and Tables

**Table 1:** Primer pairs used for qPCR.

Gene	Primer Sequences	Accession Number
*Avp*	f: AGGAGAACTACCTGCCCTCG r: AAACCCTCTCGACACTCGG	NM_016992.2
*Cyp11a1*	f: ACTTCCTGAGGGAGAACGGC r: TCCATGTTGCCCAGCTTCTC	NM_017286.3
*Cyp21a1*	f: ACGATCATCATCCCCAACAT r: CACAGCCAAAGGTCGGTATT	NM_057101.2
*Crh*	f: AACCTCAACAGAAGTCCCGC r: ACACGCGGAAAAAGTTAGCC	NM_031019.1
*Gapdh*	f: CAACTCCCTCAAGATTGTCAGCAA r: GGCATGGACTGTGGTCATGA	NM_017008.4
*Hsd11b1*	f: AAATACCTCCTCCCCGTCCT r: TTTCTCTTCCGATCCCTTTG	NM_017080.2
*Hsd11b2*	f: CAAACCCTTCCCCCACAG r: GGCTGGGCTTTTCTTAACAG	NM_017081.2
*Hsd3b1*	f: GACAGGAGCAGGAGGGTTTGTGG r: CTCCTTCTAACATTGTCACCTTGGCCT	NM_001007719.3
*Scarb1*	f: GCTTCTGGTGCCCATCATTTAC r: AGCTTGGCTTCTTGCAGTACC	NM_031541.1
*Star*	f: AGCAAGGAGAGGAAGCTATGC r: GGCACCACCTTACTTAGCACT	NM_031558.3

**Table 2:** Antibodies used for Western blot.

Antibody	Source and Type	Manufacturer	Dilution
β-actin	Mouse, monoclonal	Sigma ^1^, A5316	1:5000
StAR	Rabbit, polyclonal	Gift from Prof. Vimal Selvaraj [27]	1:500
P450scc	Rabbit, polyclonal	Protein Tech ^2^, 13363-1-AP	1:2000
3β-HSD	Mouse, monoclonal	Santa Cruz ^3^, sc-515120	1:500
Anti-mouse IgG, HRP-conjugated	Donkey, polyclonal	Santa Cruz ^3^, sc-2314	1:5000
Anti-rabbit IgG, HRP-conjugated	Donkey, polyclonal	Santa Cruz ^3^, sc-2313	1:5000

^1^ St. Louis, MO, USA; ^2^ Rosemont, IL, USA; ^3^ Dallas, TX, USA

**Table 3:** Parameters of EAE in males and females.

	Males	Females
Day of disease onset	11.07 ± 0.15	10.55 ± 0.17 #
Duration of symptoms (days)	9.23 ± 0.56	9.44 ± 0.79
Duration of paralysis (days)	1.88 ± 0.29	1.41 ± 0.29
Maximal severity score	3.18 ± 0.07	2.97 ± 0.10 #
Cumulative disease severity	14.64 ± 0.93	13.00 ± 1.17

The results are presented as mean ±SEM (*n* = 96 for males and *n* = 84 for females for day of disease onset; *n* = 34 for males and *n* = 27 for females for all the other EAE parameters). Octothorpes indicate the differences between males and females. # *p* < 0.05, Mann–Whitney test.

**Table 4:** Adrenal weight and volumetric measures of adrenal glands in males and females.

		Adrenal Weight (mg)	Adrenal Volume (mm^3^)	Medulla Volume (mm^3^)	Cortex Volume (mm^3^)	zF + zR Volume (mm^3^)	zG Volume (mm^3^)
Males	Control	30.82 ± 0.77	7.32 ± 0.74	0.68 ± 0.05	6.63 ± 0.71	5.55 ± 0.66	1.09 ± 0.06
	Onset	36.75 ± 1.44 **	7.90 ± 0.41	0.68 ± 0.07	7.22 ± 0.38	6.20 ± 0.32	1.02 ± 0.08
	Peak	39.80 ± 1.19 **	10.00 ± 0.48 *	1.04 ± 0.13	8.96 ± 0.36 *	7.61 ± 0.31 *	1.34 ± 0.06
	End	29.77 ± 0.75	7.21 ± 0.10	0.79 ± 0.05	6.43 ± 0.14	5.30 ± 0.13	1.12 ± 0.05
Females	Control	41.67 ± 1.21 ##	10.34 ± 0.37 #	0.92 ± 0.08	9.42 ± 0.37 #	7.97 ± 0.30 #	1.45 ± 0.07 #
	Onset	47.40 ± 1.80 ##	10.34 ± 0.53	0.72 ± 0.03	9.52 ± 0.32	8.42 ± 0.46 #	1.27 ± 0.10
	Peak	48.73 ± 1.02 ** ##	11.77 ± 1.33	0.85 ± 0.14	10.92 ± 1.20	9.37 ± 0.99	1.55 ± 0.28
	End	40.40 ± 2.44 ##	10.07 ± 1.24 #	0.90 ± 0.21	9.17 ± 1.03 #	7.75 ± 0.80 #	1.41 ± 0.27

The results are presented as mean ± SEM (*n* = 5 animals/group for adrenal weight, *n* = 3 animals/group for volumetric measures). The asterisks indicate the differences compared to Control within the animals of the same sex. * *p* < 0.05, ** *p* < 0.01; octothorpes indicate the differences between male and female rats of the corresponding group (Control, Onset, Peak, or End). # *p* < 0.05, ## *p* < 0.01; Kruskal–Wallis test followed by uncorrected Dunn’s post hoc test.

## Data Availability

The data are available from the corresponding author upon reasonable request.

## Supplementary Materials

The following supporting information can be downloaded at: https://www.mdpi.com/article/10.3390/biom14081020/s1, Figure S1. Original blot images presented in Figure 6. Table S1. Mean values for gene expression analyses presented in Figure 2, Figure 5 and Figure A2.
