# Supplementary material for: Alterations in the Hypothalamic–Pituitary–Adrenal Axis as a Response to Experimental Autoimmune Encephalomyelitis in Dark Agouti Rats of Both Sexes
Source: Biomolecules. 2024 Aug 17;14(8):1020. doi: 10.3390/biom14081020 (PMC11352252; doi:10.3390/biom14081020)
Supplement: Supplementary file 1 [file biomolecules-14-01020-s001.zip › Additional File S1.pdf]

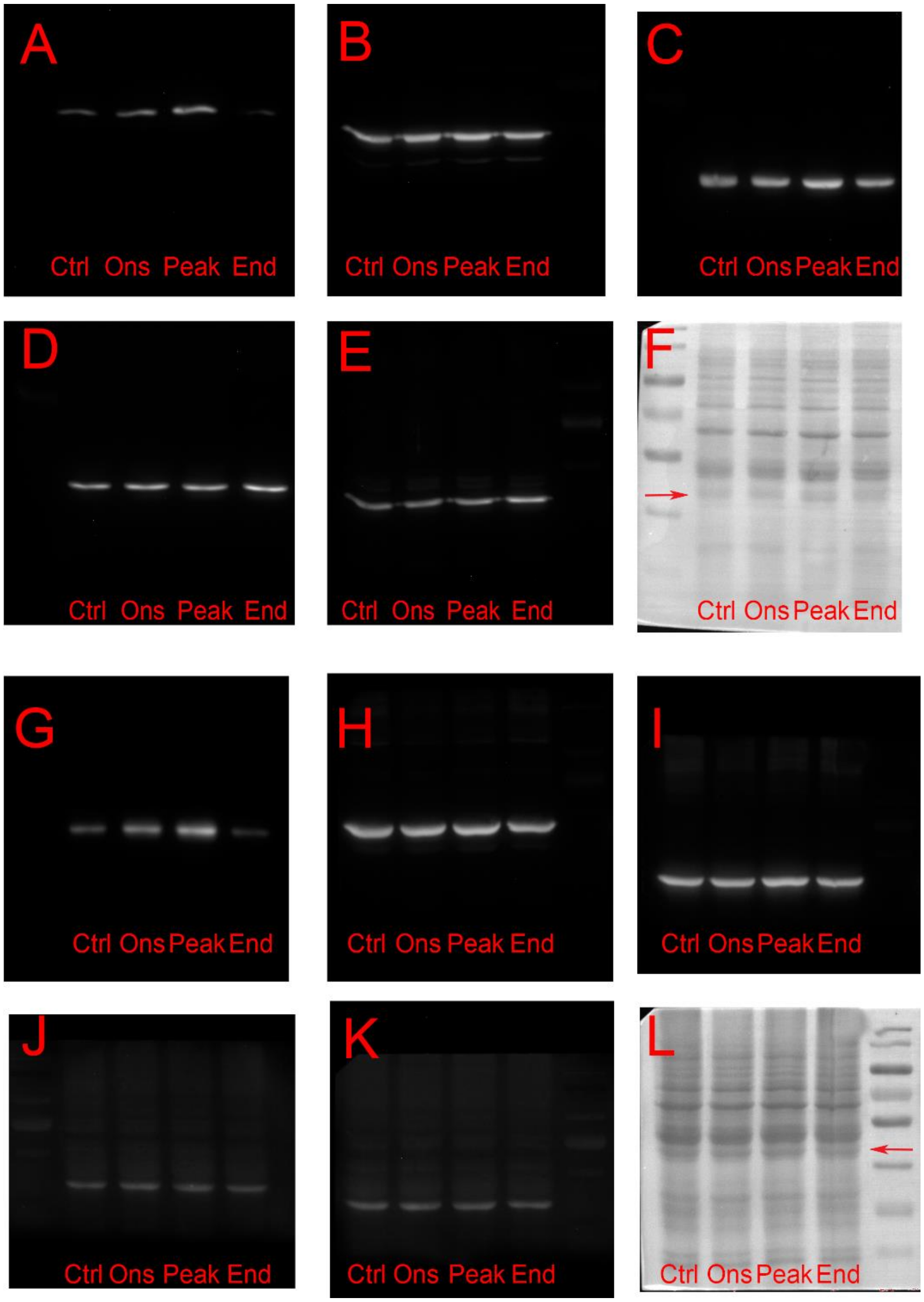

Additional file 1. Original blot images presented in Figure 6. (A-F) Males: (A) StAR, (B) P450scc, (C) 3β-HSD, (D) β-actin for StAR; (E) β-actin for P450scc; (F) total protein stain for 3 β-HSD (the arrow indicates ~42 kDa for 3β-HSD); (G-L) Females: (G) StAR, (H) P450scc; (I) 3β-HSD; (J) β-actin for StAR; (K) β-actin for P450scc; (L) total protein stain for 3β-HSD (the arrow indicates ~42 kDa for 3β-HSD). Lanes are indicated as: Ctrl – Control group; Ons – Onset group; Peak – Peak group; End – End group.
