# Supplementary material for: Alterations in the Hypothalamic–Pituitary–Adrenal Axis as a Response to Experimental Autoimmune Encephalomyelitis in Dark Agouti Rats of Both Sexes
Source: Biomolecules. 2024 Aug 17;14(8):1020. doi: 10.3390/biom14081020 (PMC11352252; doi:10.3390/biom14081020)
Supplement: Supplementary file 1 [file biomolecules-14-01020-s001.zip › Additional File S2.pdf]

	Males				Females			
	**Control**	**Onset**	**Peak**	**End**	**Control**	**Onset**	**Peak**	**End**
Avp	14.22 ± 1.82	23.98 ± 2.29	18.71 ± 2.88	24.5 ± 6.21	7.36 ± 0.75	8.14 ± 0.63	10.33 ± 1.02	9.87 ± 1.57
Crh	0.048 ± 0.006	0.032 ± 0.005	0.048 ± 0.008	0.054 ± 0.006	0.059 ± 0.004	0.037 ± 0.004	0.062 ± 0.006	0.053 ± 0.003
Cyp11a1	1091.7 ± 22.0	1450.2 ± 76.4	1558.2 ± 86.8	1078.4 ± 59.7	1333.7 ± 109.6	1688.4 ± 87.8	1847.6 ± 136.4	1536.1 ± 177.8
Cyp11b1	327.01 ± 13.36	446.73 ± 18.16	494.68 ± 19.89	312.23 ± 13.69	466.10 ± 14.33	483.54 ± 30.96	373.60 ± 18.22	524.37 ± 19.28
Cyp21a1	1836.0 ± 250.0	2396.9 ± 292.8	2381.5 ± 239.4	1522.3 ± 202.1	1095.8 ± 95.9	1353.2 ± 82.3	1344.6 ± 97.6	1344.5 ± 127.2
Hsd11b1	4.12 ± 0.17	2.62 ± 0.13	2.47 ± 0.23	3.83 ± 0.31	3.22 ± 0.25	2.35 ± 0.29	2.63 ± 0.38	4.36 ± 0.46
Hsd11b2	9.28 ± 0.56	6.21 ± 0.93	6.86 ± 0.98	8.47 ± 0.52	6.84 ± 1.05	4.43 ± 0.81	8.92 ± 1.37	9.46 ± 1.26
Hsd3b1	1284.2 ± 57.8	1210.0 ± 35.4	1335.5 ± 53.1	1094.6 ± 34.6	1064.9 ± 80.0	1047.3 ± 49.1	1117.8 ± 55.6	1224.3 ± 61.7
Scarb1	496.0 ± 55.2	1189.0 ± 168.9	1244.0 ± 135.9	258.0 ± 38.2	278.4 ± 41.9	666.4 ± 50.7	616.4 ± 92.4	347.2 ± 45.3
Star	888.2 ± 43.7	1072.0 ± 51.5	1168.0 ± 45.9	800.1 ± 58.9	998.8 ± 97.0	1136.0 ± 60.1	1381.0 ± 109.7	1010.0 ± 93.3

Additional file 2. Mean values for gene expression analyses presented in Figures 2, 5 and A2. Presented as mean ± SEM, relative to *Gapdh.*
